# Cigarette Smoking among Economically Disadvantaged African-American Older Adults in South Los Angeles: Gender Differences

**DOI:** 10.3390/ijerph16071208

**Published:** 2019-04-04

**Authors:** Shervin Assari, James L. Smith, Marc A. Zimmerman, Mohsen Bazargan

**Affiliations:** 1 Department of Family Medicine, College of Medicine, Charles R Drew University of Medicine and Science, Los Angeles, CA 90059, USA; Jamessmith@cdrewu.edu (J.L.S.); mobazarg@cdrewu.edu (M.B.); 2 Department of Health Behavior and Health Education, University of Michigan School of Public Health, Ann Arbor, MI 48109-2029, USA; marcz@umich.edu; 3 Departments of Family Medicine, University of California, Los Angeles (UCLA), Los Angeles, CA 90059, USA

**Keywords:** African Americans, Blacks, older adults, gender, depression, drinking, smoking

## Abstract

The current study aims to explore gender differences in the risk of cigarette smoking among African-American (AA) older adults who live in economically disadvantaged urban areas of southern Los Angeles. This cross-sectional study enrolled 576 older AA adults (age range between 65 and 96 years) who were residing in Service Planning Area 6 (SPA 6), one of the most economically challenged areas in southern Los Angeles. All participants had cardiometabolic disease (CMD). Data were collected using structured face-to-face interviews. Demographic factors (age and gender), socioeconomic status (educational attainment and financial difficulty), health (number of comorbid medical conditions and depressive symptoms), and health behaviors (current alcohol drinking and current smoking) were measured. Logistic regressions were used to analyze the data without and with interaction terms between gender and current drinking, depressive symptoms, and financial difficulty. AA men reported more smoking than AA women (25.3% versus 9.3%; *p* < 0.05). Drinking showed a stronger association with smoking for AA men than AA women. Depressive symptoms, however, showed stronger effects on smoking for AA women than AA men. Gender did not interact with financial difficulty with regard to current smoking. As AA older men and women differ in psychological and behavioral determinants of cigarette smoking, gender-specific smoking cessation interventions for AA older adults who live in economically deprived urban areas may be more successful than interventions and programs that do not consider gender differences in determinants of smoking. Gender-tailored smoking cessation programs that address drinking for AA men and depression for AA women may help reduce the burden of smoking in AA older adults in economically disadvantaged urban areas. Given the non-random sampling, there is a need for replication of these findings in future studies.

## 1. Introduction

Smoking is the single most important preventable behavioral risk factor of early mortality in the United States [1]. Smoking is linked to depression [2] and poor quality of life [3]. Smoking increases risk of several chronic medical conditions (CMCs), particularly cardiometabolic disease (CMD), such as diabetes, hypertension [4], heart disease [5], and stroke [6]. Smoking also increases the risk of other chronic conditions, such as cancer [7]. Smoking is linked to risk factors for cancers of the lungs [8], breasts [9], cervix [10], pancreas [7], stomach [11], and mouth [8]. As a result, smoking is a predictor of premature mortality [1]. For older adults, smoking also increases the risk of infections (e.g., pneumonia) and cognitive decline (e.g., dementia) [12].

Given the close link between CMD and smoking [4,5,6], there is a particular need to study economic, psychological, and behavioral factors that contribute to the risk of continuation of smoking in individuals who have developed comorbid medical conditions (CMCs), particularly CMD. The association between CMD and smoking is two-sided. First, smoking increases the risk of CMCs and CMD, including diabetes mellitus (DM), heart disease, stroke, and other conditions [13,14,15,16,17]. Second, individuals may decide to quit smoking when they are diagnosed with a new CMC or CMD [18,19]. This is in part because doctors advise patients with CMD who smoke to quit. While the former results in a positive association between CMD and smoking [13,14,15,16,17], the latter is reverse [18,19]. 

Gender is a strongest determinant of substance use and smoking [20,21,22,23,24]. Overall, men have a greater tendency than women to use several substances, such as tobacco and alcohol [20,21,22,23,24]. Some of the gender/sex differences in substance use are due to biology [25,26,27,28,29]. Social processes also have a major role in explaining some of the differences in substance use between men and women [30,31,32,33,34,35,36]. Due to traditional gender roles, society has different behavioral expectations from women and men. As a result, women may view substance use as less acceptable for them than men [30,31,32,33,34,35,36]. 

In addition, depression and depressive symptoms are associated with substance use [37]. Researchers have reported a very close link between depression and substance use disorder, and many individuals with depression use substances. Called a “dual diagnosis” [38], a large proportion of individuals with depression also use substances such as cigarettes, drugs, and alcohol [39]. In fact, some people may turn to substance use as a way of coping with their psychological pain, mood problems, depression, anxiety, and intense emotions. 

Finally, smoking and alcohol use tend to co-occur. One reason behind this pattern is that some substances operate as a gateway to the other ones, and some social networks and attitudes and social situations promote various types of substance uses across types. The combination of alcohol use and smoking has a considerable impact on the incidence and outcomes of CMCs and CMD [40].

Trajectories, patterns, and risk factors of smoking seems to differ among African-Americans (AA) compared to other racial and ethnic groups [41,42,43]. Smoking initiation, for example, differs between AAs and whites [43]. Minority stress theory posits that increased stress among minorities result in unhealthy behavioral coping like smoking. Predatory and targeted advertisement of menthol cigarettes, the high density of tobacco outlets, and less access to cessation programs in economically deprived urban areas expose AA communities to additional vulnerability to smoking and the use of other substances [44,45,46,47,48,49,50,51]. As a result of a phenomenon called the telescoping effect, use of tobacco and other substances are more likely to result in undesired trajectories of several negative health outcomes. As a result, AA smokers experience worse consequences, a pattern that is also shown for other substances, including alcohol [44,45,46,47,48,49,50,51,52,53,54,55,56,57,58,59,60,61,62,63,64]. Due to such a trajectory, despite a lower prevalence of substance use, AAs become vulnerable to smoking and the use of other substances [44,45,46,47,48,49,50,51,52,53,54,55,56,57,58,59,60,61,62,63,64]. 

Unfortunately, limited epidemiological knowledge exists on gender differences in the pattern and correlates of smoking among AA older adults with CMD in economically disadvantaged urban settings. Such urban settings are limited in resources like tobacco cessation programs [41,65]. Understanding more nuances on how male and female AA older adults differ in risk and protective factors that shape their smoking is very relevant for designing and implementing gender-specific smoking cessation programs in urban settings [65]. This information becomes even more relevant to AA older adults with CMD who continue to smoke. Lack of such information limits our ability to design and implement tailored interventions with the highest levels of efficacy. 

### Aims

Built on a data set that had collected data about AAs in one of the poorest areas of South Los Angeles [66,67,68,69], we explore the direct and indirect role of gender on smoking among economically disadvantaged AA older adults with CMD. Specifically, we tested the following hypotheses: (1) gender will be associated with smoking, with AA men being more likely to smoke cigarettes compared to AA women; (2) gender will moderate the co-occurrence of drinking and smoking, with the association between drinking and smoking being stronger for AA men than AA women; (3) gender will modify the association between depressive symptoms and smoking, so that depressive symptoms will show a larger effect on the prevalence of smoking of AA women than AA men; and (4) gender will modify the relevance of financial difficulty for smoking for AAs, with financial difficulty having a smaller effect on the prevalence of smoking for AA women than AA men. We focused on AAs with CMD, as most AA older adults above age 65 have at least one chronic disease, given the racial disparities in prevalence of CMCs in the United States [70]. We were also interested in understanding which AA older adults continue to smoke cigarettes despite having CMD, given the recent literature on racial variation in the effects of being diagnosed with a CMC on smoking behaviors [71]. 

## 2. Materials and Methods

### 2.1. Design and Setting

The study was a cross-sectional survey of AA older adults performed in South Los Angeles between 2015 and 2018 [66,67,68,69]. Structured face-to-face interviews were conducted between 2015 and 2018 to collect data on demographic factors (age and gender), socioeconomic status (SES; educational attainment and financial difficulty), health status (number of chronic conditions and depressive symptoms), and health behaviors (current alcohol drinking and current smoking).

### 2.2. Institutional Review Board (IRB)

The study protocol was approved by the institutional review board (IRB) of the Charles R. Drew University of Medicine and Science (CDU; IRB no. 14-12-2450-05). All participants signed a written informed consent before being enrolled in the study. Participants received financial incentive. To ensure that participants understood the consent process, participants were asked the following questions: (1) what is your understanding of the purpose for this study? (2) What is going to happen in this study? (3) What do you have to do in this study? (4) What are some risk/benefits of participating in this study? Participants enrolled to the study only if they could provide reasonable answer to the above questions.

### 2.3. Participants

The study used a non-random sampling to recruit AA older adults. Participants were a convenience sample of AA older adults residing in Service Planning Area 6 (SPA 6) in South Los Angeles. Participants were eligible if they were AA or Black, were 55 years or older, and could complete an interview in English. Participants were excluded if they were institutionalized, were concurrently enrolled to other clinical trials, or had severe cognitive impairement. As the primary study was conducted on medication challenges, it was important to limit the sample to the individuals who were mentally capable and were responsible for taking their medications. This was because our outcome was adherence, and we needed individuals who were not dependent on other individuals to take medications. Concurrent enrollment in other clinical trials was considered an exclusion criterion, because the other trials’ protocol, curriculum, and interventions could result in arbitrary changes in various participants’ physical and mental health outcomes. Only seven individuals were excluded because of concurrent participation in another clinical trial. 

This sampling resulted in 740 AAs aged 55 years and older. This sample was selected from areas in South Los Angeles, such as the Watts area. The current analysis was limited to AA participants who were 65 years or older (*n* = 576) and had CMD, including hypertension, diabetes, heart disease, and lipid disorder/hypercholesterolemia.

Participants were recruited from 11 senior housing apartment units, 16 predominantly AA churches, and low-income public housing projects located in SPA 6 in Los Angeles County. Church leaders and housing apartment managers facilitated and encouraged participation of the individuals in their communities. We recruited community-dwelling, underserved, older AAs from predominantly AA churches located in SPA 6 of Los Angeles (LA) County. LA County is the most populous county in the nation. We selected SPA 6 because the vast majority of older adults in it are AA (49%). Overall, from 10.3 million individuals who reside in LA County, more than 1.3 million are older adults (i.e., 65 years and older) [35]. Due to its large size (4300 square miles), LA County is divided into eight Service Planning Areas (SPAs) [36]. These distinct regions allow the LA Department of Public Health to better conduct surveillance, as well as provide public health and clinical services that are targeted to the specific health needs of the residents in each SPA. Approximately 28% of SPA 6 households are below the federal poverty level, and 36% of adults are uninsured. In SPA 6, 58% of adults have income levels less than 200% of the federal poverty line (FPL), compared to 41% in LA County overall. From 2013 to 2015, the percentage of homeless AAs in SPA 6 has nearly doubled from 39% to 70% [72]. 

### 2.4. Measures

#### 2.4.1. Demographic Characteristics

Age and gender were the demographic variables in this study. Age was treated as an interval variable. Gender was a dichotomous variable. Gender was the effect modifier (male 1, female 0).

#### 2.4.2. Educational Attainment

Education attainment was operationalized as an interval level variable (years of schooling). Higher scores indicated more years of education. 

#### 2.4.3. Financial Difficulty

Self-reported (perceived) financial difficulty was measured using three items that asked about the frequency with which the participant did not have enough money to afford (1) adequate food, (2) clothing, and (3) difficulty paying bills. Each item was on a response scale ranging from 1 (never) to 5 (always). A total “financial difficulty” score was calculated, ranging from 3 to 15, with a high score reflecting more financial difficulty. The Cronbach alpha of the measure in this study was 0.92. These items were consistent with Pearlin’s list of main chronic financial difficulties experienced by low SES individuals [73].

#### 2.4.4. Health Insurance

Participants were asked if they had health insurance, which was coded as a dichotomous variable (0 = no; 1 = yes).

#### 2.4.5. Comorbid Medical Conditions (CMC)

Participants were asked about 11 comorbid conditions. Individuals were asked if a physician had ever told them that they have any of these conditions: hypertension, diabetes, heart disease, lipid disorder/hypercholesterolemia, thyroid disorder, cancer, asthma, osteoarthritis, chronic obstructive pulmonary disease (COPD), rheumatoid arthritis (RA), and gastrointestinal disease. Self-reported data is a valid measure to collect data on CMC [74,75]; however, some bias in estimates of this approach to measuring multi-morbidity is expected.

#### 2.4.6. Depressive Symptoms

This study used the 15-item Short Geriatric Depression Scale (GDS) to evaluate depression [76]. Responses were on a “yes” or “no” scale. A summary score was calculated, with a potential range between 0 to 15. A higher score indicated more depressive symptoms. The Short GDS has excellent reliability and validity. This measure has been extensively used to measure depression among older adults in both clinical and community settings [77,78,79,80,81,82,83,84,85,86].

#### 2.4.7. Current Alcohol Drinking

Participants were asked “Do you drink alcohol?” Drinking was operationalized as a dichotomous variable (0 = no; 1 = yes).

#### 2.4.8. Current Cigarette Smoking

Participants were asked about their smoking habits. Participants were asked if they smoke cigarettes using the following question: “How would you describe your cigarette smoking habits?” Smoking status was operationalized as a dichotomous variable (0 = not current smoker; 1 = current smoker).

### 2.5. Statistical Analysis

We used SPSS 23.0 (IBM, Armonk, New York, NY, USA) to analyze the data. For univariate analysis, we reported frequency (*n*), relative frequency (%), means, and standard deviations (SDs). For bivariate analysis, we used the Pearson correlation test (zero order correlation) to generate a matrix of bivariate correlations between all study variables. We also used the independent samples *t*-test and Pearson’s chi-squared test to compare AA men and AA women for study variables. We applied logistic regression models with current smoking as the dependent variable, and gender, age, SES, CMCs, depressive symptoms, and current alcohol drinking as independent variables. At the first step, we performed diagnostics to check the assumptions needed for logistic regression models. We ruled out any collinearity between our independent variables. Model 1 tested the main effect of gender. Model 2 tested the gender differences in the effect of drinking on smoking. This model included an interaction between gender (female = 0; male = 1) and drinking (no = 0; yes = 1). Model 3 tested differential effects of depressive symptoms on smoking of AA men and women. This model included an interaction between males and depressive symptoms. The next model tested the gender differences in the effects of financial difficulty on smoking. This model included interaction terms between gender and education and financial strain. Finally, we reported two logistic regressions specific to genders. We reanalyzed our data using ever smoking and frequency of drinking for sensitivity analysis. As the results did not change, detailed results are not shown but are available upon request. We reported the odds ratio (OR), standard error (SE), 95% confidence intervals (95% CI), and *p*-values. 

### 2.6. Missing Data

Missing data were very limited in the main study, as well as in our current analysis. We have explained this in our methods (missing data). Our outcomes did not have any missing data. From our predictors, most variables did not have missing data. CMC data were missing for three participants, depressive symptoms were missing for one participant, and financial difficulty was missing for two participants.

## 3. Results

### 3.1. Descriptive Statistics

Table 1 describes the study variables in the sample. All participants were older adults (65 years or older) with at least one CMD. From all our participants, 40.1% (*n* = 231) had DM, 96.5% (*n* = 556) had hypertension (HTN), 15.8% (*n* = 91) had stroke, and 33.9% (*n* = 195) had heart disease. From all our participants, 99% (*n* = 570) had some type of health insurance. Participants were mostly females (65.6%). Our sample had an average age of 74 years old. AA women were slightly older than AA men in our sample. From our participants, 29.5% reported current alcohol drinking, and 14.8% reported current cigarette smoking. 

### 3.2. Bivariate Analysis

Table 2 shows the results of bivariate correlations between study variables. This table reports correlation coefficients (*r*) based on Pearson correlation. Current smoking and current drinking statuses were positively correlated (*r* = 0.27, *p* < 0.05), suggesting that participants who report current smoking where more likely to drink alcohol as well. This correlation was larger for AA men (*r* = 0.43, *p* < 0.05) than for AA women (*r* = 0.12, *p* < 0.05). Depressive symptoms and smoking were positively correlated in the pooled sample (*r* = 0.17, *p* < 0.05), suggesting that participants who report higher levels of depressive symptoms also report smoking. This correlation was, however, larger for AA women (*r* = 0.26, *p* < 0.05) than AA men (*r* = 0.06, *p* > 0.05). 

### 3.3. Multivariable Analysis

Table 3 shows the results of several multiple logistic regression models with smoking as the outcome. Model 1 tested the main effect of gender, without any interaction terms in the model. Model 2 tested the gender differences in the effect of drinking on current smoking. Model 3 tested differential effect of depressive symptoms on the current smoking of AA men and women. Model 4 tested the gender differences in the roles of two SES indicators on the odds of current smoking. 

Model 1 showed that male gender is associated with greater odds of current smoking (OR = 2.85, 95% CI = 1.67–4.86). In this model, older age was associated with lower odds of current smoking (OR = 0.87, 95% CI = 0.82–0.92). In addition, depressive symptoms (OR = 1.14, 95% CI = 1.03–1.26) and drinking alcohol (OR = 3.61, OR = 2.13–6.13) were associated with greater odds of current smoking in the pooled sample (Table 3, Model 1).

Table 3 also reports the results of Model 2, which includes the gender by drinking interaction term. This model shows a positive interaction between gender (men) and drinking, suggesting that the effect of drinking alcohol on current smoking is larger for men than women. 

This table also shows the results of Model 3, which includes the gender and depressive symptoms interaction term. This model shows a negative interaction between gender (men) and depressive symptoms, suggesting that the effect of depressive symptoms on current smoking is smaller for men than women. 

Table 3 also shows the results of Model 4, which includes the gender and financial distress interaction term. This model shows no interaction between gender and financial distress, suggesting no gender difference in the effects of financial difficulty on current smoking. 

Table 4 shows the results of Models 5 and 6, which tested logistic regressions specific to genders. The results indicate that depressive symptoms were a predictor of the odds of smoking for women, but not men. The association between drinking and current smoking, however, was not statistically significant at the 0.05 level for women. Yet this association (i.e., drinking and current smoking) was statistically significant for men (Table 4).

**Figure 1:**
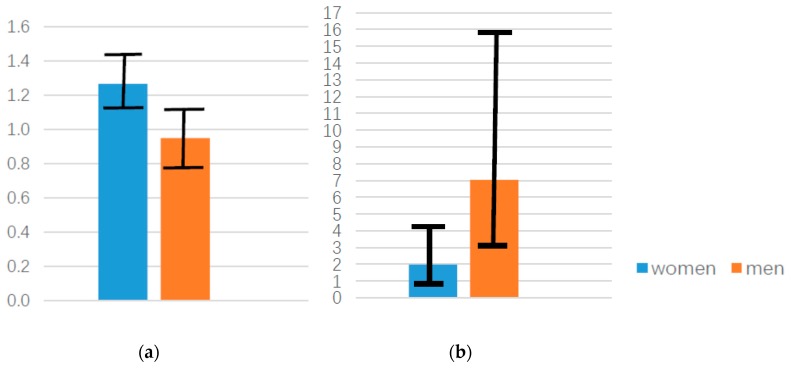
Gender differences in the odds ratio (OR) of high depressive symptoms (**a**) and current drinking (**b**).

Figure 1a shows a larger OR, reflecting the effects of depressive symptoms on current smoking for women than men. As this figure shows, the 95% CI does not cross 1 for women; however, 1 is included in the 95% CI for men. Figure 1b shows a larger OR, reflecting the effects of current drinking status on current smoking for men than women. As this figure shows, the 95% CI does not cross 1 for men; however, 1 is included in the 95%CI for women. 

## 4. Discussion

In a sample of AA older adults in economically deprived urban areas of South Los Angeles, the current study showed three main findings. First, AA older men are more likely than AA older women to smoke cigarettes. Second, alcohol may have a larger association with smoking for AA older men than AA older women. Third, depressive symptoms are more closely associated with the smoking of AA older women than AA older men. As the sample of this study was not random, the results should be considered suggestive. 

Our results supported our hypothesis that among older AA adults who live in economically challenged urban areas that are limited in resources, men are more likely to be current smokers than women. This was in line with what we know about role of gender on substance use and externalizing behaviors. Men learn in society that they can express themselves with externalizing behaviors, while women sometimes feel obligated to comply with traditional norms and societal expectations. As a result, men are more likely to show risky behaviors, such as smoking and drinking [87,88,89,90,91,92,93,94,95]. Another reason for this robust gender difference is that men systemically underestimate, while women over-estimate risk across domains [88,96,97,98,99,100,101].

The results also support our hypothesis that depressive symptoms play a more salient role on the smoking of elderly AA women than men. This finding suggests that smoking may be used differently as a coping mechanism for AA older men and women with CMD. To be more specific, for AA older women, smoking may be a way to deal with depression; however, for AA older men, depression may not similarly be linked to smoking. AA women may be more likely than AA men to use smoking as a coping mechanism to reduce their psychological distress and depression. 

Older age was associated with less smoking. Age is a strong and consistent determinant of substance use [32,102]. Overall, younger people have a higher tendency to smoke cigarette and drink alcohol [32,102]. Biological mechanisms may explain the lower tendency of older adults to use substances [25,26,27,28,29]. Older adults have lower metabolism (biological tolerance) for tobacco and alcohol [25], which may result in lower likelihood of use. Cohort differences may also explain some of the differences in smoking between age groups. Popularity of smoking in some eras and the availability of information about risks associated with various substances differ across cohorts and age groups [103,104,105,106]. 

Another finding of this study was that smoking in AA older adults with CMD was not influenced by subjective or objective SES indicators. In addition, financial distress did not affect the smoking of AA older adults with CMD. This is in contrast to researchers who have found that subjective indicators of SES (e.g., financial difficulty) are particularly consequential for both AAs [107,108,109] and older adults [110,111,112]. Research has indicated that financial difficulty comes with a higher risk of health consequences and health needs [113], such as smoking [114]. Growing evidence, however, suggests that the health implications of SES indicators are smaller for AAs than whites [41,115,116,117,118,119,120]. As suggested by the Minorities Diminished Returns (MDR) theory [121], SES indicators have a smaller effect on AAs than on whites. Although most of this research is focused on a comparison of AAs and whites [41,115,116,117,118,119,120], some research shows that even within AAs, AA men may gain less health from SES than AA women [122]. AA men with high educational attainment and income report poor mental health [122], a pattern which is not shown for AA women. For example, high educational attainment reduces the symptoms of depression and psychological distress for AA women, but not AA men [76]. Thus, AA men may be at a relative disadvantage compared to AA women regarding the mental health gain from their SES resources. 

### 4.1. Limitations

The current study had a few limitations. First, it used a non-random sample. As a result, findings are not generalizable to all African-American older adults in the United States. Second, due to the cross-sectional design, inference of causal links between variables is not possible. There is a need for longitudinal studies that explore gender differences in using smoking as a coping strategy over time. The third limitation was the measurement of smoking, drinking, and CMCs. Our indicators to measure smoking and drinking were single items, which reduces the validity of our measures, and limited the variation of our outcome variables. Future research may use more comprehensive measures capable of increasing the validity of our measures and capturing greater variance of the outcome. Our measurement of smoking was too simplistic. We conceptualized smoking as a dichotomous variable based on the question, “How would you describe your cigarette smoking habits?” This classification may be partially biased, as it is not clear if all responses of “still smoking” have the same meaning. Some people may report “still smoking” if they smoked today, and some may report current smoking if they smoked last week. There is also a need for similar studies with individuals from other racial and ethnic groups. Such research can inform us how racial and ethnic populations differ in smoking, and determinants of such behaviors. Such research may provide additional insight into racial and ethnic heath disparities. We did not have data on the cognitive impairment of our participants. Future research may use standardized measures like the Mini-Mental State Exam (MMSE). Finally, income may be a very important SES indicator. Unfortunately, we did not have access to the income of the participants. Despite these significant limitations, the study still adds to our knowledge of a section of the population that is not well studied. Another possible source of bias is relying on self-report data for CMCs. Self-reported data about CMCs can be validated using administrative data. The findings, however, still provide evidence that smoking may have different predictors in AA men and AA women. We found support for our hypothesized gender differences. Both the hypotheses and the results of this study are important from a public health perspective. However, the non-random sample may be considered a major limitation for this study. The potential policy implication of this study is therefore compromised by the restricted study sample. This is mainly because the external validity of this study is low. In addition, our measurement of smoking was too simplistic. 

### 4.2. Implications

The finding that the co-occurrence of the health problems of drinking, depressive symptoms, and smoking tend to differ in older AA men and women in economically disadvantaged urban areas has implications for program planning. One implication is the need for the provision and expansion of tobacco cessation and mental health care services in poor urban areas. We argue that the integration of depression care and smoking may be more relevant for AA older women than men, while integration of alcohol and tobacco programs may particularly benefit AA older men. Thus, the content of behavioral interventions could differ for male and female AA older adults. 

## 5. Conclusions

Gender impacts the smoking prevalence of AA older adults with CMD who reside in economically deprived urban areas in multiple ways. First, gender has a primary effect on smoking, with men having a higher tendency to smoke than women. However, the effect of gender is not limited to a higher risk of smoking in men than women. Smoking seems to accompany drinking more commonly in men than women, while depression seems to have a larger effect on smoking of women than men. That means that smoking and drinking tend to co-occur in AA older men, while depression and smoking tend to co-occur in AA older women, when both groups live in economically challenged urban areas. Integration of tobacco and alcohol prevention programs may generate more benefits for AA older men with CMD than AA older women with CMD. In addition, smoking cessation programs that screen for depression may be a useful approach for older AA women.

## Figures and Tables

**Table 1:** Descriptive statistics.

Characteristics	All	Women	Men
	Mean (SD)	Mean (SD)	Mean (SD)
Age (Years)	74.06 (6.92)	74.46 (6.91)	73.30 (6.89)
Educational Attainment (Years) *	12.70 (2.33)	12.95 (2.02)	12.23 (2.76)
Financial Strain	8.21 (4.92)	8.11 (4.67)	8.39 (5.38)
Comorbid Medical Conditions	2.97 (1.83)	2.08 (1.83)	2.74 (1.80)
Depressive Symptoms	2.10 (2.44)	2.08 (2.45)	2.16 (2.43)
	***n* (%)**	***n* (%)**	***n* (%)**
Gender			
Women	378 (65.6)	378 (100.0)	-
Men	198 (34.4)	-	198 (100.0)
Current Drinking Status *			
No	406 (70.5)	277 (73.3)	129 (65.2)
Yes	170 (29.5)	101 (26.7)	69 (34.8)
Current Smoking Status *			
No	491 (85.2)	343 (90.7)	148 (74.7)
Yes	85 (14.8)	35 (9.3)	50 (25.3)

* *p* < 0.05 for comparison of men and women.

**Table 2:** Bivariate correlations.

Characteristics	1	2	3	4	5	6	7	8
**All**								
1 Gender (Male)	1.00	−0.08	−0.15 **	0.03	−0.09 *	0.02	0.09 *	0.21 **
2 Age (Years)		1.00	−0.19 **	−0.10 *	0.01	−0.10 *	−0.14 **	−0.26 **
3 Educational Attainment (Years)			1.00	−0.14 **	−0.08 *	−0.07	0.05	−0.03
4 Financial Difficulty				1.00	0.23 **	0.31 **	0.16 **	0.16 **
5 Comorbid Medical Conditions					1.00	0.33 **	0.00	0.05
6 Depressive Symptoms						1.00	0.07	0.17 **
7 Current Drinking							1.00	0.27 **
8 Current Smoking								1.00
**Women**								
2 Age (Years)		1.00	−0.22 **	−0.11 *	0.02	−0.12 *	−0.08	−0.16 **
3 Educational Attainment (Years)			1.00	−0.05	−0.12 *	−0.06	0.07	0.04
4 Financial Difficulty				1.00	0.25 **	0.32 **	0.14 **	0.16 **
5 Comorbid Medical Conditions					1.00	0.31 **	−0.03	0.06
6 Depressive Symptoms						1.00	0.04	0.26 **
7 Current Drinking							1.00	0.12 *
8 Current Smoking								1.00
**Men**								
2 Age (Years)		1.00	−0.20 **	−0.08	−0.04	−0.06	−0.24 **	−0.39 **
3 Educational Attainment (Years)			1.00	−0.23 **	−0.07	−0.09	0.05	−0.03
4 Financial Difficulty				1.00	0.21 **	0.28 **	0.20 **	0.15 *
5 Comorbid Medical Conditions					1.00	0.38 **	0.07	0.09
6 Depressive Symptoms						1.00	0.13	0.06
7 Current Drinking							1.00	0.43 **
8 Current Smoking								1.00

* *p* < 0.05, ** *p* < 0.01.

**Table 3:** Summary of multivariable logistic regression models in the pooled sample.

	OR	95% CI	OR	95% CI	OR	95% CI	OR	95% CI
	Model 1		Model 2		Model 3		Model 4	
Gender (Men)	2.85 ***	1.67–4.86	1.69	0.80–3.58	5.60 ***	2.61–12.00	3.90 **	1.41–10.80
Age (Years)	0.87 ***	0.82–0.92	0.87 ***	0.82–0.92	0.87 ***	0.82–0.92	0.87 ***	0.82–0.92
Educational Attainment	0.96	0.86–1.07	0.96	0.85–1.07	0.96	0.86–1.07	0.95	0.85–1.07
Financial Difficulty	1.03	0.98–1.08	1.03	0.98–1.08	1.03	0.98–1.08	1.05	0.98–1.12
Comorbid Medical Conditions	1.02	0.88–1.19	1.02	0.87–1.18	1.03	0.89–1.20	1.02	0.87–1.19
Depressive Symptoms	1.14 *	1.03–1.26	1.14 *	1.03–1.26	1.25 ***	1.11–1.42	1.13 *	1.02–1.26
Current Drinking (Yes)	3.61 ***	2.13–6.13	2.13 *	1.00–4.51	3.72 ***	2.18–6.36	3.63 ***	2.14–6.16
Gender (Men) × Current Drinking	-	-	2.89 *	1.00–8.41	-	-	-	-
Gender (Men) × Depressive Symptoms	-	-	-	-	0.79 *	0.65–0.95	-	-
Gender (Men) × Financial Difficulty	-	-	-	-	-	-	0.97	0.88–1.06

Outcome: Smoking (Current). * *p* < 0.05, ** *p* < 0.01, *** *p* < 0.001.

**Table 4:** Summary of multivariable logistic regression models by gender.

	OR	95% CI	OR	95% CI
	Women (Model 5)		Men (Model 6)	
Age (Years)	0.92 *	0.86–0.99	0.81 ***	0.74–0.89
Educational Attainment	1.07	0.86–1.35	0.88 #	0.76–1.02
Financial Difficulty	1.05	0.98–1.13	1.01	0.93–1.09
Comorbid Medical Conditions	0.97	0.78–1.21	1.14	0.91–1.44
Depressive Symptoms	1.27 ***	1.12–1.43	0.95	0.79–1.14
Current Drinking (Yes)	1.97 #	0.91–4.23	7.03 ***	3.10–15.91

Outcome: Smoking (Current). # *p* < 0.1, * *p* < 0.05, *** *p* < 0.001.
